# PCDD/Fs and DL-PCBs in Chinese Mitten Crab (*Eriocheir sinensis*) and Its Farming Environment in Shanghai, China

**DOI:** 10.3390/foods11172556

**Published:** 2022-08-24

**Authors:** Ya Li Wang, Si Ying Fei, Tian Wei Wang, Xue Ting Liu, Xiao Nin Gao, Hao Tian Wu, Kun Hu

**Affiliations:** 1 National Demonstration Center for Experimental Fisheries Science Education, Shanghai Ocean University, Shanghai 201306, China; 2 National Pathogen Collection Center for Aquatic Animals, Shanghai Ocean University, Shanghai 201306, China; 3 Key Laboratory of Freshwater Aquatic Genetic Resources, Ministry of Agriculture, Shanghai Ocean University, Shanghai 201306, China

**Keywords:** persistent organic pollutant, PCDD/Fs, DL-PCBs, high-resolution gas chromatography and high-resolution mass spectrometry (HRGC/HRMS), *Eriocheir sinensis*, bioaccumulation

## Abstract

Most polychlorinated dibenzo-p-dioxins and dibenzofurans (PCDD/Fs) and dioxin-like polychlorinated biphenyls (DL-PCBs) in the human body are acquired from dietary intake. The chronic exposure of humans to PCDD/Fs and DL-PCBs is a major health concern, and these compounds are strictly controlled in many areas. This study measured the levels of PCDD/Fs and DL-PCBs in Chinese mitten crab (*Eriocheir sinensis*) farms in Shanghai and determined potential sources. The mean concentrations of PCDD/Fs and DL-PCBs in the studied crab samples were 264.20 ± 260.14 and 506.25 ± 226.80 pg/g ww (wet weight), respectively. The range of the toxic equivalent (TEQ) for the total PCDD/Fs and DL-PCBs in the crab samples was 1.20–29.04 pg TEQ/g ww. Further analysis revealed that the TEQ input to crabs in aquacultural water was 1.6 times higher than the TEQ in edible crab parts. Aquatic plants, shore plants, and feed contributed about 0.05% of the total TEQ input to crabs. The TEQ contribution from sediment was 317 times that found in edible crab parts, and sediment may be the most prevalent source of PCDD/Fs and DL-PCBs in farm crabs. The evaluation of the Shanghai market crab revealed different levels of PCDD/Fs and DL-PCBs. The TEQs for the mean PCDD/F and DL-PCB levels were 1.55 ± 1.96 and 1.05 ± 0.55 pg TEQ/g ww, respectively. The tolerable daily intake (TDI) levels of adults and children were lower than the prescribed range (1–4 pg TEQ/kg (weight)·d), indicating no significant chronic or acute ingestion risk for adults and children.

## 1. Introduction

Polychlorinated dibenzo-p-dioxins and dibenzofurans (PCDD/Fs) are a group of halogenated aromatic hydrocarbons that exhibit high toxic potential and that include polychlorinated dibenzo-dioxins (PCDDs) and polychlorinated dibenzo-furans (PCDFs), comprising a total of 210 congeners [1]. They typically accumulate in the air, soil, sediment, fish, human adipose tissue, and milk [2,3]. Dioxin-like polychlorinated biphenyls (DL-PCBs) are persistent organic pollutants that remain in the environment for long periods of time [4]. They are hailed as the most hazardous elements for human health and were enlisted during the Stockholm Convention owing to their high carcinogenicity [5]. DL-PCBs have a lipophile and stable chemical structure, making them resistant to degradation [6]. They are introduced into the aquatic environment, tend to settle in the sediment, enter the food chain via numerous routes, and accumulate in living organisms [7]. The International Agency for Research on Cancer (IARC) has classified PCDD/Fs and DL-PCBs as “Group I human carcinogens “ [8]. As inducements, PCDD/Fs and DL-PCBs have become research hotspots. Previous studies revealed that the by-products of industrial manufacturing processes involving chlorine remain the major sources of PCDD/Fs and DL-PCBs, which are strongly associated with natural heat [9]. Subsequently, their footprints have also been found in the North and South Poles and may play a significant role in migration processes, posing a serious threat to the earth’s ecological environment [10,11]. In the atmosphere, PCDD/Fs and DL-PCBs may be transported into the ocean and rivers via environmental deposition and may affect the health of aquatic animals. The PCDD/Fs and DL-PCBs are widely distributed in the aquatic environment and accumulate in aquatic organisms, being integrated into the food chain [12]. Hyo-bang Moon et al. [13] recorded the highest levels of PCDD/Fs and DL-PCBs residues in crustaceans followed by fish. Dietary intake is believed to be the main route of human exposure to dioxins, accounting for more than 90% of total human exposure [5]. They may be present in one’s diet, as it is possible for them to be passed up through the food chain and to accumulate in humans at the top. At the same time, the presence of these compounds in the human body leads to serious health concerns due to their toxicity, indirectness, stability, and generational hereditary. They can also interfere with the immune, reproductive, and nervous systems and render a carcinogenic impact on human beings [6]. Therefore, food safety issues related to dioxins are attracting more and more attention. The tolerable daily intake (TDI) levels recommended by the World Health Organization (WHO) at a 1990 dioxin conference in the Netherlands were 10 pg TEQ/(kg (weight)·d) [14]. Since then, following the emergence of new epidemiological data and data regarding the significant effects of dioxins on neurological development and the endocrine system, the WHO experts consulted these data and re-evaluated their stance on dioxins, determining a new allowable TDI range of 1–4 pg TEQ/(kg (weight)·d) to ensure the safety of food and to build health barriers [15].

The Chinese mitten crab (*Eriocheir sinensis*) is a common freshwater crab, accounting for 18% of shrimp crab culture. They are widely distributed from Northern China to South Korea, existing in freshwater as juveniles and adults. Upon reaching sexual maturity, this crab migrates to brackish water to mate, lay eggs, and hatch, and it then returns to its freshwater habitat [16]. Its delicious meat, richness in unsaturated fatty acids, unique pleasant aroma, and delicious taste have promoted the Chinese mitten crab as a popular aquatic product. In 2021, global sales of the Chinese mitten crab market reached hundreds of millions of dollars, providing a substantial advantage to the marine industry. However, according to Taiwan’s Food and Drug Administration, in October 2018, Taiwan had imported about 196 tons of Chinese mitten crab from the mainland, 40 tons of which contained more dioxin than standard levels, accounting for 20% of the total product (https://www.mohw.gov.tw/cp-16-44846-1.html (accessed on 21 January 2022). The sampling limit was four times higher than the maximum of 27 pq/g TEQ/g ww (more than 6.5 pq/g TEQ/g (wet weight)). In October 2017, the dioxin content of Chinese mitten crab from Hunan province exceeded the standard limit. Among them, 4.114 tons of hairy crabs constituted 4.1 pg/g of dioxin, and 4.455 tons of hairy crabs were found to constitute 12.1 pg/g of dioxin and PCBs, seriously affecting crab sales (https://www.ereying.com/articles/83.html (accessed on 27 January 2022).

The accumulation of contaminants in aquatic organisms is closely related to the food chain and its culture environment [17]. Studies have shown that animal feed remains one of the primary sources of pollution, and contaminated animal feed or feed additives may lead to food contamination [18,19]. In 1999, dioxin contamination was found in chicken feed in Belgium [20]; in the USA, livestock, aquaculture, and poultry feed exceeded dioxin limits from 2002 to 2003, and this revealed that dioxins enter the food chain via feed, potentially contributing to dioxin accumulation [21]. Other studies have shown that soil is the source of dioxin contamination in eggs from free-range hens on Polish farms [22]. As omnivorous animals, mixed feed is the primary source of nutrition for Chinese mitten crabs. Moreover, Chinese mitten crabs also eat aquatic plants and benthic animals. Contaminants in sediment might be the source of dioxins in samples of these crabs [23]. Some substances, such as water bodies, food residues, and feces, will eventually form sediments, which may lead to dioxin accumulation and contamination in sediments and the development of secondary pollution [24]. Sediment is also a feeding route for crabs, so the chemical constituents in sediment can affect crab health. For Chinese mitten crab, the cultural environment has gained gradual prominence in its growth and development. The wide variety of substances consumed by Chinese mitten crabs indicates that the sources of dioxins and biphenyls may be complex and that the food and culture environment of Chinese mitten crabs may be contributing factors.

Currently, the sources of PCDD/Fs and DL-PCBs found in Chinese mitten crab in Shanghai are not clearly understood. This study investigated the PCDD/F and DL-PCB contents in Chinese mitten crabs and the potential sources of these contaminants. The amount of pollution found in Chinese mitten crab on the market was analyzed to provide a scientific basis for targeted prevention and control measures, public scientific consumption, and essential support to ensure the healthy breeding and sustainable development of livestock, poultry, and aquatic products.

## 2. Materials and Methods

### 2.1. Crab Sampling

**Figure 1:**
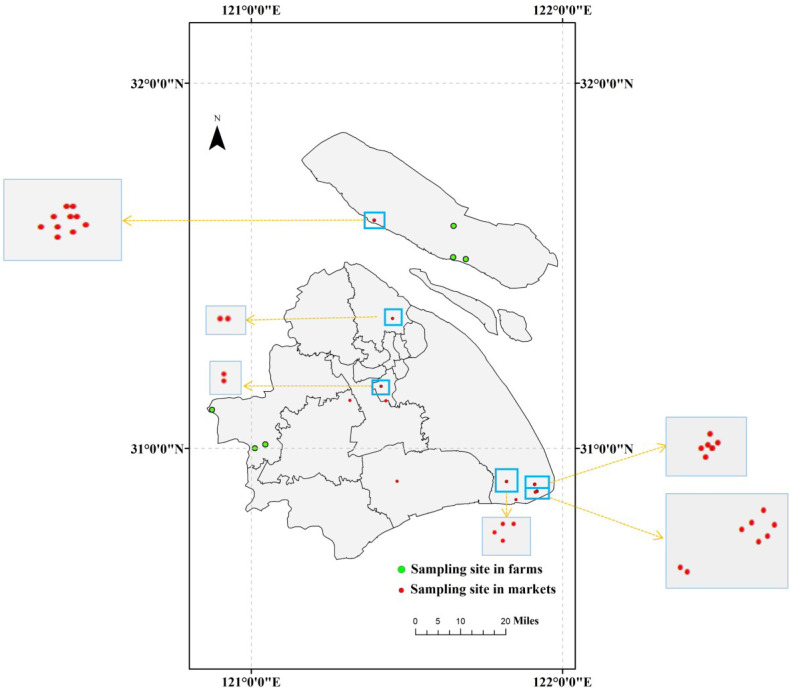
Crab sampling sites in Shanghai.

Chinese mitten crabs were obtained from various farms situated in the Qingpu and Chongming districts of Shanghai (Table 1). Among the sampling sites, three were in the Qingpu district and three were in the Chongming district (Figure 1). A total of 90 crabs were collected, 15 at each sampling site, and each crab was the commercial standard size of about 100.2 ± 5.6 g. In addition, Chinese mitten crab samples sold in fresh markets around Shanghai were also procured for further testing and evaluation. A total of 555 crabs were collected, 15 at each sampling site, and each crab was about 101.6 ± 3.6 g. The sampling information for the market crabs is shown in Table S1 and Figure 1. After arriving at the lab, the samples were freeze-dried and stored in a refrigerator at −20 °C.

### 2.2. Sampling of Potential Sources of PCDD/Fs and DL-PCBs

To determine the primary sources of the PCDD/Fs and DL-PCBs found in Chinese mitten crabs, various crab culture processes, including sediment (-CJW), culture water (-YS), aquatic grass (-SC), shore plants (-ZW), and feed (-SL), were collected. We collected 6 replicates of sediment samples from 6 farms (each not less than 1 kg dw). Comprehensive samples were taken from 6 different locations with the crab farms’ pool aquaculture water (every 20 L), feed samples (each not less than 500 g ww), aquatic plants (each not less than 1 kg ww), and shore plants (each not less than 1 kg ww). The corresponding farm information is shown in Table 1.

### 2.3. PCDD/Fs and DL-PCBs Detection Method

The chromatographic method used in this experiment is currently the internationally recognized standard method for dioxin detection and analysis and mainly uses a combination of high-resolution gas chromatography and high-resolution mass spectrometry (HRGC/HRMS) [25]. This study adopts the method outlined by the National Food Safety Standard for the determination of toxic equivalent of dioxins and their analogs in food (GB 5009.205-2013) for sample analysis [26]. High-resolution gas chromatography, high-resolution mass spectrometry, and a gas chromatography column (HRGC-HRMS, Agilent, Palo Alto, CA, USA) with a resolution of no less than 10,000 were used in this experiment. The contents of 17 2,3,7,8-substituted polychlorinated dibenzo-dioxins (PCDDs) and polychlorinated dibenzo-furans (PCDFs) and 12 dioxin-like polychlorinated biphenyls (DL-PCBs) and their toxic equivalents (TEQs) were analyzed. Details of these 29 compounds are shown in Table S2. Quantitative internal standard: ^13^C_12_-labeled solution of the internal standard for the quantification of PCDD/Fs and PCBs compounds; internal standard of recovery: ^13^C_12_-labeled solution of internal standard for the recovery of PCDD/F and PCB compounds (Wellington Laboratory, Guelph, ON, Canada). Details of these internal standards are shown in Tables S3 and S4.

### 2.4. Sample Preparation

The collected samples were packaged in light-protected aluminum foil and brown glass bottles, transported to the laboratory at the Shanghai Academy of Agricultural Sciences at a low temperature, and stored at −20 °C. The collected water samples were adsorbed on a resin chromatography column containing XAD-2 (Serva, Sparks, NV, USA) and were stored in the dark after drying. Before sample processing, the edible parts, sediments, feed, aquatic plants, and shore plant samples that were collected from the Chinese river crab farms were crushed and ground in a freeze dryer (Pilot-4LD, Beijing Biocool, Beijing, China). The samples were mixed well with the appropriate amount of internal standard before extraction (details of quantitative internal standard in Table S3). After that, using accelerated solvent extraction (Dionex ASE300, Thermo Fisher Scientific, Waltham, MA, USA)), 200 mL of n-hexane: dichloromethane (1:1, volume ratio) (GR, Sigma, Ronkonkoma, NY, USA) was used for solvent extraction. All of the extracts produced from the feed and crab samples needed to be pickled 3 times with 30 mL of concentrated sulfuric acid (GR, Sinopharm Chemical, Beijing, China) to remove any lipids. They also needed to then be rinsed with 50 mL of deionized water three times to neutralize the acidity, and anhydrous sodium sulfate (GR, Kermel, Tianjin, China) was then used on the samples to remove moisture. The samples were then set aside. Finally, all of the extracts were concentrated using a rotary evaporator (R-210V, Buchi, Hendrik-Ido-Ambacht, Sweden), and the concentrates were purified using a series of adsorption chromatography columns, such as acidic silica gel columns, composite silicone columns, and alkaline alumina columns (Thermo, Waltham, MA, USA). The eluate of each component obtained after purification and separation was concentrated from 1 mL to 2 mL using a rotary evaporator and by increasing the nitrogen concentration (Organomation, Berlin, MA, USA), and the eluates were then transferred to a sample bottle filled with a 0.2 mL lined tube and concentrated to about 20 μL, and the appropriate amount of internal standard required for recovery was added to each component (the amounts of internal standard required for recovery are shown in Table S4) for on-machine detection.

### 2.5. Instrumentation and Measurements

The PCDD/F and DL-PCB contents in the samples were determined using the coupled HRGC-HRMS method. Different GC columns should be used for different targets. Detailed information regarding the chromatographic conditions is shown in Table 2.

The GC conditions were optimized to achieve high sensitivity with rapid and accurate separation. After condition optimization, the same GC conditions were optimized for standard solution, blank, IPR and OPR, and sample determination. The mass spectrometer was tuned using a reference gas (PFK or FC43) to monitor m/z 330.9792 or other PFK fragment ions in the m/z 300 to 350 mass range to achieve a mass spectrometer resolution of 10,000 (10% peak-to-valley). During the study, the amount of reference gas entering the HRMS was regulated to ensure that the chosen signal intensity of the locking mass number did not exceed 10% of the detector’s whole range. The mass shift of the mass spectrometer was corrected using the PFK’s (or another reference gas’) locking mass number. The method of adding recovery rate internal standard was used to perform quality control on the analysis and determination of the samples, and a certain amount and standard solution were added before the samples were processed to determine the recovery rate to correct the error caused by the losses incurred during sample processing and the change in the sample amount during analysis. We excluded test results that were not included in the recovery rate to ensure the reliability of the results. At the same time, blank experiments were performed during sample handling to prevent contamination.

### 2.6. Statistical Analysis

Toxicity equivalent (TEQ) calculations are based on the revised WHO-TEF values from 2005. All of the TEQ concentrations are reported according to upper, median, and lower limits. The TEQs of the PCDD/Fs and DL-PCBs in Chinese mitten crab were calculated according to Formulas (1)–(6):

***TEQ****_i_* = ***TEF***** × *c****_i_* (1)

***TEQ****_PCDDs_* = Σ***TEF****_iPCDDs_* × ***c**_iPCDDs_* (2)

***TEQ****_PCDFs_* = Σ***TEF****_iPCDDFs_* × ***c**_iPCDFs_* (3)

***TEQ****_PCDD/Fs_* = ***TEQ**_PCDDs_* + ***TEQ**_PCDFs_* (4)

***TEQ****_DL-PCBs_* = Σ***TEF****_iDL-PCBs_* × ***c**_iDL-PCBs_* (5)

***TEQ****_(PCDD/Fs_*_ + *DL-PCBs)*_ = TEQ*_PCDD/Fs_* + TEQ*_DL-PCBs_* (6)

where *TEQ_i_*—dioxin toxic equivalent (*TEQ*) of the congeners of *PCDD/Fs* or *DL-PCBs* in food in micrograms per unit kg (μg/kg); *TEF_i_*—toxicity equivalent factors of congeners in PCDD/Fs or DL-PCBs; *c_i_*—concentrations of *PCDD/Fs* or *DL-PCBs* congeners in food in micrograms per kilogram (μg/kg); the remaining subscripts are specific combinations of *PCDD/Fs* or *DL-PCBs*.

## 3. Results and Analysis

### 3.1. The Concentration of PCDD/Fs and DL-PCBs in Farm Crabs

**Figure 2:**
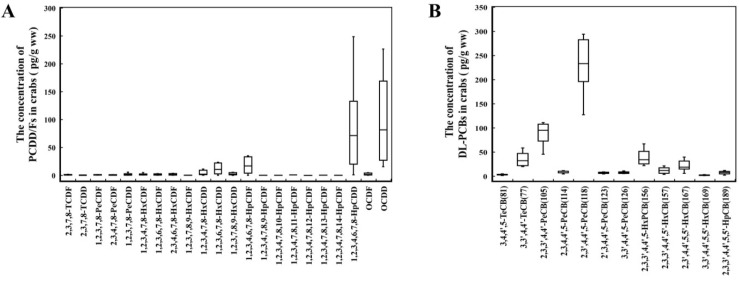
Concentrations of PCDD/Fs (**A**) and DL-PCBs (**B**) in farmed Chinese mitten crabs (*n* = 90).

**Figure 3:**
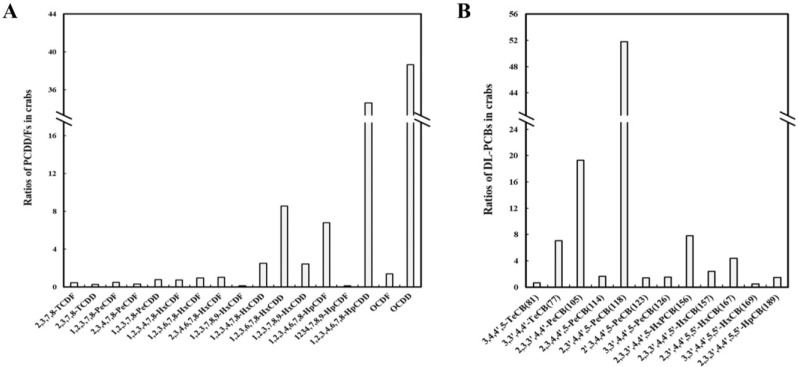
Ratios of PCDD/Fs (**A**) and DL-PCBs (**B**) in farmed Chinese mitten crabs.

A total of 17 PCDD/Fs and 12 DL-PCBs were detected in the edible parts of the sampled crabs, and the PCDD/F and DL-PCB concentrations in the crab samples from the different farms varied greatly. Detailed results are shown in Table S5. The mean PCDD/F and DL-PCB concentrations of the crab samples were 264.20 ± 260.14 and 506.25 ± 226.80 pg/g ww, respectively. The PCDD/F concentrations ranged from 20.45 to 686.64 pg/g ww, with OCDD having the greatest concentration (612.75 pg/g ww), accounting for 38.7%, followed by 1,2,3,4,6,7,8-HpCDD at 549.05 pg/g ww, accounting for 34.6% (Figure 2A and Figure 3A). The DL-PCB concentrations ranged from 246.51 to 894.47 pg/g ww, with the highest concentration of 2,3′,4,4′,5-PeCB (1573.97 pg/g ww) accounting for 51.8%, followed by 2,3,3′,4,4′-PeCB (86.63 pg/g ww), which accounted for 19.8% (Figure 2B and Figure 3B). The remaining PCDD/F and DL-PCB congeners in the crab samples accounted for less than 10%.

The TEQs of the total PCDD/F and DL-PCB levels in the crab samples ranged from 1.20 to 29.04 pg TEQ/g ww, and the mean was 9.37 pg TEQ/g ww. Similarly, the TEQ range for the PCDD/Fs was 0.54–27.86 pg TEQ/g, and the mean was 8.50 pg TEQ/g ww; the TEQ range for the DL-PCBs was 0.60–1.18 pg TEQ/g ww, and the mean was 0.87 pg TEQ/g ww. The TEQs of the PCDD/Fs and DL-PCBs in the crabs from three farms (MH, RJ, and NXC) were higher than the standard limits (3.5 pg/g for PCDD/Fs and 6.5 pg/g for the total PCDD/Fs and dl-PCBs) [27]. The TEQs of the PCDD/Fs and DL-PCBs in the farm crab samples are shown in Supplementary Table S7. 

### 3.2. The Concentration of PCDD/Fs and DL-PCBs in Market Crabs

**Figure 4:**
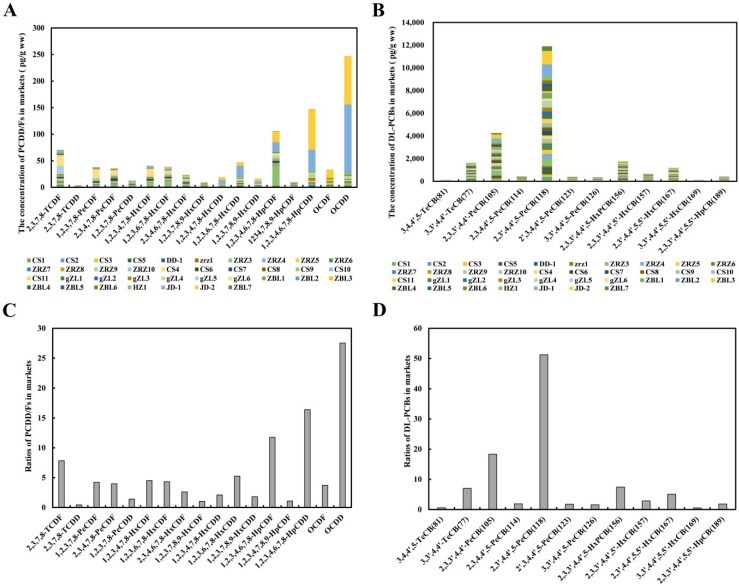
Concentrations of PCDD/F (**A**) and DL-PCB (**B**) in Chinese mitten crabs from markets (*n* = 555). PCDD/F (**C**) and DL-PCB (**D**) ratios in Chinese mitten crabs from markets.

The PCDD/F and DL-PCB contents in 37 crab samples from fresh markets in Shanghai were determined, and the specific results are shown in Table S8. The concentration range and average level of PCDD/Fs in these crab samples were 223.62–1934.88 and 24.27 ± 53.05 pg/g ww, respectively. The concentration range and mean values of the DL-PCBs were 228.85–2163.32 pg/g ww and 628.19 ± 375.48 pg/g ww, respectively. OCDD (375.48 pg/g ww) and 1,2,3,4,6,7,8-HpCDD (3.98 pg/g ww) were the most abundant PCDD/Fs. For the DL-PCBs, 2,3′,4,4′,5-PeCB (321.68 pg/g ww) and 2,3,3′,4,4′-PeCB (115.10 pg/g ww) exhibited the highest abundance (Figure 4).

The PCDD/F and DL-PCB detection rates in the market crab samples were 100%, and in some samples, the TEQ ranges of the PCDD/Fs and DL-PCBs exceeded the relevant limits [27]. The total PCDD/F and DL-PCB TEQ levels in the market crab samples ranged from 0.33 to 10.80 pg TEQ/g, and the mean was 2.67 pg TEQ/g. The total PCDD/F TEQs ranged from 0.11 to 8.80 pg TEQ/g, and the mean was 1.56 pg TEQ/g. The total DL-PCB TEQs ranged from 0.23 to 2.35 pg TEQ/g, and the mean was 1.11 pg TEQ/g. TEQs of the total PCDD/F and DL-PCB contents in 8% of the samples were higher than the relevant limit (6.5 pg TEQ/g for the total PCDD/Fs and DL-PCBs) [27]. The TEQ of the PCDD/Fs in 14% of the samples was higher than the relevant limit (3.5 pg TEQ/g for PCDD/Fs) [27]. The TEQs of the total PCDD/Fs and DL-PCBs found in market crabs are shown in Table S6. TEFs were used to determine the TEQ distribution of individual congeners. 1,2,3,7,8-PeCDD; 2,3,4,7,8-PeCDF; and 2,3,7,8-TCDF contributed 22%, 19%, and 12%, respectively, and the TEQs of the remaining congeners accounted for less than 10%. Among the DL-PCBs, 3,3′,4,4′,5-PeCB (89%) are the main contributors to the TEQs of the DL-PCBs, followed by 3,3′,4,4′,5,5′-HxCB (8%) and 2,3′,4,4′,5-PeCB (0.8%). The toxicity equivalent factors of the 17 PCDD/Fs and 12 DL-PCBs were determined according to the WHO-TEF standards (Table S2) that were revised by WHO in 2005.

### 3.3. The Concentration of PCDD/Fs and DL-PCBs in Potential Sources

To determine the major sources of PCDD/Fs and DL-PCBs in crabs, we further examined the concentrations of PCDD/Fs and DL-PCBs in each stage of crab farming. For specific results, see Table S5. The potential sources of dioxins consist of feed, including fishmeal, soybean meal, rapeseed meal, cottonseed meal, soybean phospholipid oil, dihydrogen calcium phosphate, and other chemicals to suit the crab’s development requirements. In addition, other sources included water and plants from farmed crab ponds, plants on the shore of farmed crab ponds, and sediments at the bottom of farmed crab ponds.

Some substances in aquaculture water, such as impurities, food residues, and feces, eventually turn into sediments that may be more susceptible to contamination. The experimental results showed that the concentrations of PCDD/Fs and DL-PCBs in sediment were the highest on the farms, with concentrations ranging from 109.02 to 35,301.67 pg/g dw. The mean of the PCDD/F and DL-PCB concentrations in the sediment samples was 13,050.71 ± 14,855.84 pg/g dw. The concentration of the PCDD/Fs and DL-PCBs in aquaculture water ranged from 8.30 to 574.71 pg/L, with a mean concentration of 211.27 ± 217.07 pg/L. The concentrations of the PCDD/Fs and DL-PCBs in aquatic plants ranged from 3.28 to 419.12 pg/g ww, with a mean concentration of 139.83 ± 159.68 pg/g ww. The concentrations of the PCDD/Fs and DL-PCBs in shore plants ranged from 2.17 to 194.30 pg/g ww, with a mean concentration of 52.48 ± 76.80 pg/g ww. The concentration of the PCDD/Fs and DL-PCBs in feed ranged from 7.26 to 68.37 pg/g dw, with a mean concentration of 40.22 ± 23.19 pg/g dw.

**Figure 5:**
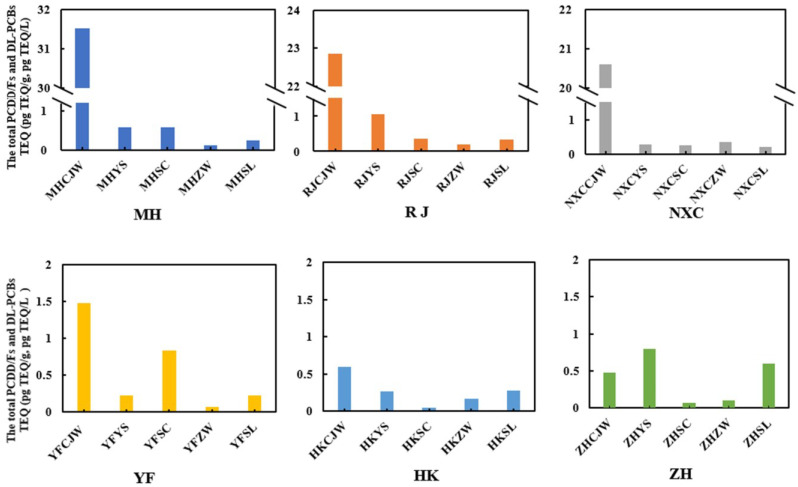
The total PCDD/F and DL-PCB TEQs in the potential sources.

The TEQs of the potential sources of PCDD/Fs and DL-PCBs are shown in Table S7. On the farms, sediments showed the highest TEQs, ranging from 0.48 to 31.51 pg TEQ/g dw. The total TEQs of the PCDD/Fs and DL-PCBs in aquaculture water, aquatic plants, and shore plants ranged from 0.23 to 1.06 pg TEQ/L, from 0.05 to 0.83 pg TEQ/g ww, and from 0.06 to 0.34 pg TEQ/g ww, respectively. The TEQs of the PCDD/Fs and DL-PCBs in feed ranged from 0.22 to 0.60 pg TEQ/g dw, in compliance with EU limit regulation EU NO 277/2012 (feed) (the maximum residue limit was 1.75 pg TEQ/g for PCDD/Fs and 5.5 pg TEQ/g for PCDD/Fs + DL-PCBs). The total TEQs of the PCDD/Fs and DL-PCBs from the three farms in Qingpu district (MH, RJ, and NXC) were greater than those found in the three farms in Chongming district (YF, HK, and ZH) (Figure 5).

The congeners of PCDD/Fs and DL-PCBs in the potential sources and in the crabs were similarly distributed. OCDD and 1,2,3,4,6,7,8-HpCDD were dominant in aquaculture water, aquatic plants, and, especially, in sediments. 2,3′,4,4′,5-PeCB was abundant in sediment, aquaculture water, and feed. Different congeners contribute differently to the TEQ in different samples. For PCDD/Fs, 1,2,3,4,6,7,8-HpCDD had the highest TEQ in sediment, aquatic plants, and aquaculture water, with contribution rates of 39%, 32%, and 21%, respectively. In addition, in feed, 1,2,3,7,8-PeCDD contributed to 40%. For the DL-PCBs, 3,3′,4,4′,5-PeCB made the most dominant contributions in almost all of the samples, followed by 3,3′,4,4′,5,5′-HxCB, which is consistent with our conclusion that 3,3′,4,4′,5-PeCB (90%) and 3,3′,4,4′,5,5′-HxCB (9%) predominate in crab samples.

### 3.4. Bioaccumulation of PCDD/Fs and DL-PCBs in Crabs

Sediments, aquacultural water, aquatic plants, shore plants, and feed can all enter crabs, so pollutants from each aquacultural process can accumulate in crabs, posing a threat to our health. The above results showed that the DL-PCBs to PCDD/Fs concentration ratio in farm crabs was 191.62%. In sediments, aquacultural water, aquatic plants, shore plants, and feed, the DL-PCB to PCDD/F concentration ratios were 0.20%, 2.72%, 2.22%, 13.36%, and 2553.51%, respectively. In contrast, the concentrations of PCDD/Fs were higher in sediments, aquacultural water, aquatic plants, and shore plants than in crabs, while the concentrations of DL-PCBs were lower in sediments, aquacultural water, aquatic plants, and shore plants than in crabs (Figure S1). These results indicate that crabs bioaccumulate DL-PCBs more readily than PCDD/Fs and that raw materials used in feed may be contaminated with DL-PCBs.

**Figure 6:**
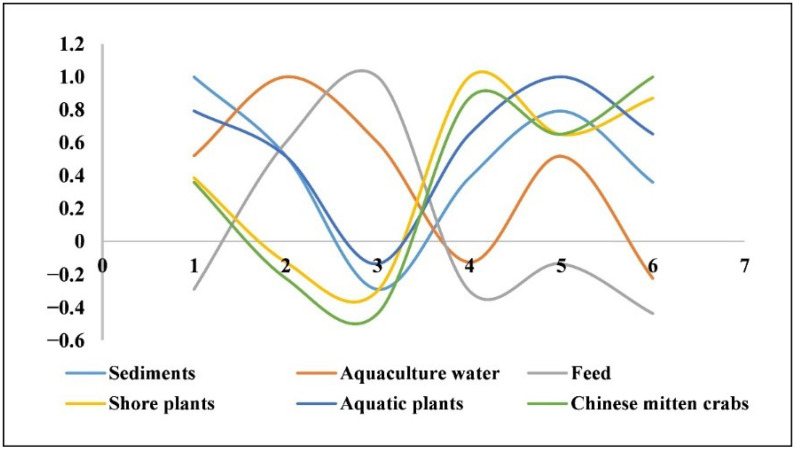
Correlation analysis of the residual pollutants in Chinese mitten crabs and the potential sources.

To determine the key link responsible for the accumulation of pollutants in crabs, we conducted a correlation analysis of the residual pollutants in crabs and the potential sources, and the correlation coefficient R was used to measure the relationship between the crabs and potential sources. The correlation coefficient between crabs and sediment was 0.89 (R > 0.8), showing a strong correlation, while the correlation coefficient between crabs and aquaculture water was 0.28, showing a weak correlation and suggesting that the accumulation of PCDD/Fs and DL-PCBs in crabs in aquaculture mainly comes from sediments (Figure 6). PCDD/Fs and DL-PCBs can enter crabs through feed, water, aquatic plants, and shore plants; remain in crab tissues through distribution and metabolism; are excreted in feces and urine; and further migrate into sediments, increasing the exposure of crabs to pollutants.

## 4. Discussion

On 27 November 2020, the International Agency for Research on Cancer (IARC) updated its latest list of carcinogens, and both 2,3′,4,4′, 5-PeCB (118) and 2,3,3′,4,4′-PeCB (105) were classified as class I carcinogens. The findings of the current study revealed that the highest proportions of PCB-118 and PCB-105 were found in farm crabs (51.8% and 19.8%) and in market crabs (51.2% and 18.3%). Compared to PCDD/Fs, DL-PCBs were abundantly produced and used in the past, and they are relatively difficult to degrade under the influence of physical and chemical factors [28]. The most decent explanation for the above statement may be that the presence of residual PCB-118 and PCB-105 in crab may make DL-PCBs more stable. In one study, Grazia Barone et al. [1] found that PCB-118 was dominant in bluefin tuna samples. Wang X. et al. [29] reported that PCB-118 and PCB-105 were predominant in all of the fish samples they studied. Similarly, Danae Costopoulou et al. [30] also found that the main congener for monoorthmic PCBs produced in Greece was PCB-118 followed by PCB-105. These results align with our experiment’s key finding, which state that PCB-118 and PCB-105 comprise the dominant concentrations and closely related to their ability to enrich organisms.

OCDD and 1,2,3,4,6,7,8-HpCDD are polychlorinated dibenzo-p-dioxins (PCDDs) as well as the dominant PCDD/F congener [31,32]. Battisti Sabrina et al. [33] found that OCDD was the most abundant PCDD/F congener in dairy products collected in the Latium region of Italy (2011–2017). L. P. Fang et al. [34] found that fish tissues contained a relatively large amount of OCDD, accounting for 54% of the total PCDD/F congeners. Our results showed that OCDD was dominant in both farm crabs (38.7%) and market crabs (12.7%), concordant with the above scholars’ conclusions, our results suggest that OCDD has a strong enrichment capacity. Milena Dömötörová et al. [35] observed that 1,2,3,4,6,7,8-HpCDD was the second most abundant congener in most of the soil samples considered in their study. Kim K.S. et al. [36] found that 1,2,3,4,6,7,8-HpCDD was predominant in all of their soil samples. Regarding this substance, Loganathan B.G. et al. [37] observed the same pattern of HpCDD accumulation in sediment and mussel tissues. Our results showed that 1,2,3,4,6,7,8-HpCDD is the second highest PCDD/F congener in crabs (34.6% in farm crabs and 10.0% in market crabs) after OCDD.

We then conducted further research on content and analyzed the sources of PCDD/Fs and DL-PCBs in crabs to search for possible sources. Our results showed that the means of the TEQs of the PCDD/Fs and PCBs in crabs, sediments, aquacultural water, aquatic plants, shore plants, and feed were 9.37 pg TEQ/g, 12.92 pg TEQ/g, 0.53 pg TEQ/L, 0.28 pg TEQ/g, 0.17 pg TEQ/g, and 0.32 pg TEQ/g. We referred to Han Ying’s [38] method to evaluate all of the substances that hairy crabs may ingest in a 1-hectare (ha) crab pond. In crab ponds, every hectare produces about 50 kg of Chinese mitten crab, and the edible portion accounts for about 26% of the crabs’ weight. As a result, the production of crab meat and roe (crab paste) per hectare of crab pond is about 13 kg/ha, implying that the PCDD/Fs and DL-PCBs TEQ in crab meat and roe (crab paste) per hectare of crab pond is approximately 122 ng TEQ/ha. The recovery rate of hairy crabs was assumed to be 70% per hectare of crab pond, and a TEQ of 52 ng was found in dead crabs. A mean feed dose of 95 kg/ha resulted in a PCDD/Fs and DL-PCBs input of 30 ng TEQ/ha. The coverage rate of aquatic plants and shore plants in crab ponds was about 50% per hectare, or about 0.265 kg; the water (aquacultural water) content at a depth of 0.8 m was about 534 m^3^; the sediment weight of the 0.1 m disturbed layer was about 4269 kg. The total TEQ exposure of PCDD/Fs and DL-PCBs was 0.12 ng/ha, 283 ng/ha, and 55,155 ng/ha. We found that the amount of PCDD/Fs and PCBs input into crab ponds is much higher than that output to crabs. The TEQ of the PCDD/Fs and DL-PCB input to crabs in aquacultural water was 1.6 times higher than the TEQ in edible crab parts. Aquatic plants, shore plants, and feed contributed about 0.05% of the total TEQ inputs to crabs. Without considering biological processes, the TEQ contribution from sediment was 195 times that from aquacultural water. Therefore, sediment is considered the major contributor to PCDD/Fs and DL-PCBs in Chinese mitten crabs. All of the external substances added in the cultivation of crab culture can settle into the sediment, affecting the historical concentration of pollutants in the sediment, increasing the exposure risk of hairy crab to pollutants.

The results showed that the total TEQ of PCDD/Fs and DL-PCBs in Shanghai’s Qingpu district, China, were significantly higher than in Shanghai’s Chongming district, China (Figure 5). This situation may be closely related to the surrounding industrial environment. The three aquaculture farms in Qingpu district were all raised by the Taipu River. Hong Yao et al. [39] found that a large amount of wastewater containing heavy metals was discharged into the Taihu Basin every year. The Taipu River, an important tributary of the Taihu Basin, is similarly plagued by wastewater contamination, which might be one of the explanations for the elevated levels of dioxins and dioxin-like PCBs in farms in the Qingpu district. To supplement the experimental results, we detected the level of pollutants in the soil around farms in the Qingpu and Chongming districts. The findings revealed that the level of pollutants in the soil near the farms in Qingpu district (4.89 pg TEQ/g dw in MH, 19.07 pg TEQ/g dw in RJ, 3.78 pg TEQ/g dw in NXC) was higher than that near the farms in Chongming district (1.00 pg TEQ/g dw in YF, 0.89 pg TEQ/g dw in HK, 0.86 pg TEQ/g dw in ZH). The pollutants migrated into crab farms over a long period of time, which further indicated that the crab farms in Qingpu district had high levels of pollutants. Detailed results are shown in Supplementary Tables S9 and S10.

Our above investigation on market crab revealed that the average residual value of dioxins and dioxin-like PCBs in crabs was 0.0027 pg TEQ/kg. Based on the average daily consumption of adults (60 kg) and children (13.1 kg) of 3 crabs and 1 crab (at an average weight of 100 g per crab), the TDIs of adults and children would be 0.0135 pg TEQ/g (weight)·d and 0.0206 pg TEQ/g (weight)·d, respectively, lower than the prescribed range, indicating no significant chronic ingestion risk for adults and children (WHO experts determined that the TDI range of total PCDD/Fs and PCBs was 1–4 pg TEQ/kg (weight)·d) [15]. The TDIs for adults and children were 0.054 pg TEQ/g (weight)·d and 0.0824 pg TEQ/g (weight)·d when calculated with the maximum residual value of 0.0108 pg TEQ/kg, respectively, both of which were below the prescribed range, indicating that there was no significant risk of acute ingestion in adults and children. Taking into account the above data analysis and the daily quota determined by the WHO, the consumption of Chinese mitten crabs does not seem to pose a threat to health. However, given the persistence and bioaccumulation of such pollutants, the excessive consumption of aquatic products may increase the burden of dioxin-type pollutants in the body. Therefore, it is recommended that the government increase its detection of pollutants, that individuals eat normal amounts of aquatic products, and that certain restrictions be imposed on the consumption of aquatic products. Although there may be some uncertainties and limitations, the study provides a valuable assessment of the health risks associated with PCDD/Fs and DL-PCBs exposure in Chinese river crabs.

## 5. Conclusions

The PCDD/F and DL-PCB contents in crabs from the market and from farms in Shanghai were investigated. It was found that crabs bioaccumulate DL-PCBs more readily than PCDD/Fs. As the main source, the total TEQ exposure to PCDD/Fs and DL-PCBs in sediments was 55,155 ng/ha. The PCDD/F and DL-PCB contents in crabs was generally safe, and crabs had a strong enrichment ability for 2,3′,4,4′,5-PeCB (118), 2,3,3′,4,4′-PeCB (105), OCDD, and 1,2,3, 4,4,6,7,8-HpCDD. A health and safety assessment based on market crab samples showed no significant chronic or acute ingestion risk for adults and children, suggesting that eating crab several times a year may not cause PCDD/Fs and DL-PCBs to exceed safe limits. Finally, by further studying the PCDD/F and DL-PCB contents in the farming process, the PCDD/Fs and DL-PCBs in crabs mainly come from sediments. Therefore, regular sediment treatment can effectively reduce the exposure of crabs to pollutants.

## Figures and Tables

**Table 1:** Farm crab sampling site information.

Name of Farm	Latitude and Longitude	Number
Shanghai Ruijie Aquaculture Professional Cooperative	31°0′40″ N, 121°2′45″ E	RJ
Nanxin Village farm	31°31’11″ N, 121°41′20″ E	NXC
Shanghai Mahe Product Professional Cooperative	31°0′1.54″ N, 121°0′44″ E	MH
Shanghai Zihao Industrial Development Company Limited	31°35′4″ N, 121°39′14″ E	ZH
Shanghai Yufeng Aquaculture Professional Cooperative	31°36′37″ N, 121°39′0″ E	YF
Shanghai Huikang Aquaculture professional cooperative	31°31′12″ N, 121°41′21″ E	HK

**Table 2:** Chromatographic condition information.

Instrumental Conditions	PCDD/Fs Detection	DL-PCBs Detection
DB-5 ms column	60 m × 0.25 mm × 0.25 μm	60 m × 0.25 mm × 0.25 μm
Inlet temperature	280 °C	290 °C
Transmission line temperature/interface temperature	310 °C	290 °C
Column temperature	120 °C (hold for 1 min); ramp up to 220 °C at 43 °C/min (hold for 15 min); ramp up to 250 °C at 2.3 °C/min, ramp up to 260 °C at 0.9 °C/min, ramp up to 310 °C at 20 °C/min (hold for 9 min)	80 °C (hold for 2 min); ramp up to 150 °C at 15 °C/min; ramp up to 270 °C at 2.5 °C/min (hold for 3 min), ramp up to 330 °C at 15 °C/min (hold for 1 min)
Carrier gas flow rate	1.2 mL/min	1.2 mL/min

## Data Availability

All available data are presented in the article.

## Supplementary Materials

The following supporting information can be downloaded at: https://www.mdpi.com/article/10.3390/foods11172556/s1, Table S1: Market crab sampling site information, Table S2: Summary of 29 compounds and WHO 2005 TEF Values, Table S3: Solutions of isotopically labelled quantitative internal standards for PCDD/Fs and DL-PCBs, Table S4: Solutions of isotopically labelled recovery internal standards for PCDD/Fs and DL-PCBs, Table S5: The concentrations of PCDD/Fs and DL-PCBs in Chinese mitten crabs and the potential sources, Table S6: PCDD/F and DL-PCB TEQs in Chinese mitten crabs from fresh markets, Table S7: The total PCDD/F and DL-PCB TEQs in Chinese mitten crabs and the potential sources, Table S8: The concentrations of PCDD/Fs and DL-PCBs in market crabs, Table S9: The total PCDD/F and DL-PCB TEQs in in the soil around Qingpu and Chongming district farms, Table S10: Informed detection limit (DL) of the method and the percentage of recovery of each PCDD/F and DL-PCB congener analyzed. Figure S1: Ratios of DL-PCBs/PCDD/Fs in farm Chinese mitten crabs and potential sources.
