# Supplementary material for: PCDD/Fs and DL-PCBs in Chinese Mitten Crab (Eriocheir sinensis) and Its Farming Environment in Shanghai, China
Source: Foods. 2022 Aug 24;11(17):2556. doi: 10.3390/foods11172556 (PMC9455688; doi:10.3390/foods11172556)
Supplement: Supplementary file 1 [file foods-11-02556-s001.zip › foods-1851665-supplementary.pdf]

foods

MDPI

Article

PCDD/Fs and DL-PCBs in Chinese Mitten Crab (*Eriocheir sinensis*) and Its Farming Environment in Shanghai, China

Ya Li Wang [1,2,3], Si Ying Fei [1,2,3], Tian Wei Wang [1,2,3], Xue Ting Liu [1,2,3], Xiao Nin Gao [1,2,3], Hao Tian Wu [1,2,3] and Kun Hu [1,2,3,*]

1 National Demonstration Center for Experimental Fisheries Science Education, Shanghai Ocean University, Shanghai 201306, China
2 National Pathogen Collection Center for Aquatic Animals, Shanghai Ocean University, Shanghai 201306, China
3 Key Laboratory of Freshwater Aquatic Genetic Resources, Ministry of Agriculture, Shanghai Ocean University, Shanghai 201306, China
* Correspondence: khu@shou.edu.cn

Table S1. Market crab sampling site information.

Sample Number	Name of Market	Latitude and Longitude
CS1	Agribusiness Supermarket (Guzong Road Store)	30.879999, 121.912126
CS2	Shangyou fresh Supermarket (Jiuxin Road Store))	31.132332, 121.317558
CS3	Luchaogang Seafood Market	30.858812, 121.85068
CS4	RT-Mart(Ni Chen Store)	30.909157, 121.819986
CS5	RT-Mart(Ni Chen Store)	30.909157, 121.819986
CS6	RT-Mart(Ni Chen Store)	30.909157, 121.819986
CS7	RT-Mart(Ni Chen Store)	30.909157, 121.819986
CS8	Big mouth fresh Supermarket (Yuxiu East Road)	31.626545, 121.39455
CS9	Walmart(Guilin Road)	31.170957, 121.417017
CS10	Walmart(Guilin Road)	31.170957, 121.417017
CS11	Ocean fresh supermarket(Xu Hui Store)	31.131148, 121.433076
DD-1	Agribusiness Supermarket (Guzong Road Store)	30.879999, 121.912126
ZRZ1	Qingcheng Vegetable Market	31.626545, 121.39455
ZRZ3	Qingcheng Vegetable Market	31.626545, 121.39455
ZRZ4	Qingcheng Vegetable Market	31.626545, 121.39455
ZRZ5	Qingcheng Vegetable Market	31.626545, 121.39455
ZRZ6	Qingcheng Vegetable Market	31.626545, 121.39455
ZRZ7	Qingcheng Vegetable Market	31.626545, 121.39455
ZRZ8	Qingcheng Vegetable Market	31.626545, 121.39455
ZRZ9	Qingcheng Vegetable Market	31.626545, 121.39455
ZRZ10	Qingcheng Vegetable Market	31.626545, 121.39455
gZL1	Guzong Road Vegetable Market	30.882815, 121.917545
gZL2	Guzong Road Vegetable Market	30.882815, 121.917545
gZL3	Guzong Road Vegetable Market	30.882815, 121.917545
gZL4	Guzong Road Vegetable Market	30.882815, 121.917545
gZL5	Guzong Road Vegetable Market	30.882815, 121.917545
gZL6	Guzong Road Vegetable Market	30.882815, 121.917545

ZBL1	Zhubai Road Vegetable Market	30.900998, 121.910281
ZBL2	Zhubai Road Vegetable Market	30.900998, 121.910281
ZBL3	Zhubai Road Vegetable Market	30.900998, 121.910281
ZBL4	Zhubai Road Vegetable Market	30.900998, 121.910281
ZBL5	Zhubai Road Vegetable Market	30.900998, 121.910281
ZBL6	Zhubai Road Vegetable Market	30.900998, 121.910281
ZBL7	Zhubai Road Vegetable Market	30.900998, 121.910281
JD-1	Jiangyang Seafood Market	31.356989, 121.453813
JD-2	Jiangyang Seafood Market	31.356989, 121.453813

Table S2. Summary of 29 compounds and WHO 2005 TEF Values.

Compound	WHO 2005 TEF
Chlorinated dibenzo-*p*-dioxins	
2,3,7,8-TCDD	1
1,2,3,7,8-PeCDD	1
1,2,3,4,7,8-HxCDD	0.1
1,2,3,6,7,8-HxCDD	0.1
1,2,3,7,8,9-HxCDD	0.1
1,2,3,4,6,7,8-HpCDD	0.01
OCDD	0.0003
Chlorinated dibenzofurans	
2,3,7,8-TCDF	0.1
1,2,3,7,8-PeCDF	0.03
2,3,4,7,8-PeCDF	0.3
1,2,3,4,7,8-HxCDF	0.1
1,2,3,6,7,8-HxCDF	0.1
1,2,3,7,8,9-HxCDF	0.1
2,3,4,6,7,8-HxCDF	0.1
1,2,3,4,6,7,8-HpCDF	0.01
1,2,3,4,6,7,8,9-HpCDF	0.01
OCDF	0.0003
Non-*ortho*–substituted PCBs	
3,3′,4,4′-tetraCB (PCB 77)	0.0001
3,4,4′,5-tetraCB (PCB 81)	0.0003
3,3′,4,4′,5-pentaCB (PCB 126)	0.1
3,3′,4,4′,5,5′-hexaCB (PCB 169)	0.03
Mono-*ortho*–substituted PCBs	
2,3,3′,4,4′-pentaCB (PCB 105)	0.00003
2,3,4,4′,5-pentaCB (PCB 114)	0.00003
2,3′,4,4′,5-pentaCB (PCB 118)	0.00003
2′,3,4,4′,5-pentaCB (PCB 123)	0.00003
2,3,3′,4,4′,5-hexaCB (PCB 156)	0.00003
2,3,3′,4,4′,5′-hexaCB (PCB 157)	0.00003
2,3′,4,4′,5,5′-hexaCB (PCB 167)	0.00003
2,3,3′,4,4′,5,5′-heptaCB (PCB 189)	0.00003

Note: TEF means toxic equivalency factor. WHO 2005 TEF was formulated to harmonize the toxic equivalency factors (TEFs) for the international level of dioxin and dioxin-like compounds.

Table S3. Solutions of isotopically labelled quantitative internal standards for PCDD/Fs and DL-PCBs.

Category	Congener name	Number
PCDD/Fs[a]	$^{13}C_{12}$-2,3,7,8-TCDF	76523-40-5
	$^{13}C_{12}$-2,3,7,8-TCDD	89059-46- 1
	$^{13}C_{12}$-1,2,3,7,8-PeCDF	109719-79- 1
	$^{13}C_{12}$-2,3,4,7,8-PeCDF	109719-77-9
	$^{13}C_{12}$-1,2,3,7,8-PeCDD	116843-02-8
	$^{13}C_{12}$-1,2,3,4,7,8-HxCDF	109719-80-4
	$^{13}C_{12}$-1,2,3,6,7,8-HxCDF	109719-81-5
	$^{13}C_{12}$-2,3,4,6,8,9-HxCDF	114423-98-2
	$^{13}C_{12}$-1,2,3,7,8,9-HxCDF	116843-03-9
	$^{13}C_{12}$-1,2,3,4,7,8-HxCDD	116843-04-0
	$^{13}C_{12}$-1,2,3,6,7,8-HxCDD	116843-05- 1
	$^{13}C_{12}$-1,2,3,4,6,7,8-HpCDF	109719-83-7
	$^{13}C_{12}$-1,2,3,4,7,8,9-HpCDF	109719-84-8
	$^{13}C_{12}$-1,2,3,4,6,7,8-HpCDD	109719-94-0
	$^{13}C_{12}$-OCDD	114423-97- 1
DL-PCB[b]	$^{13}C_{12}$-3,4,4',5-TePCB	81L
	$^{13}C_{12}$-3,3',4,4'-TePCB	77L
	$^{13}C_{12}$-2,3,3',4,4'-PePCB	105L
	$^{13}C_{12}$-2,3,4,4',5-PePCB	114L
	$^{13}C_{12}$-2,3',4,4',5-PePCB	118L
	$^{13}C_{12}$-2',3,4,4',5-PePCB	123L
	$^{13}C_{12}$-3,3',4,4',5-PePCB	126L
	$^{13}C_{12}$-2,3,3',4,4',5-HxPCB	156L
	$^{13}C_{12}$-2,3,3',4,4',5'-HxPCB	157L
	$^{13}C_{12}$-2,3',4,4',5,5'-HxPCB	167L
	$^{13}C_{12}$-3,3',4,4',5,5'-HxPCB	169L
	$^{13}C_{12}$-2,3,3',4,4',5,5'-HpPCB	189L

Note: [a] number is the CAS login number. [b] is the International Union of Pure Applied Chemistry (IUPAC) code.

Table S4. Solutions of isotopically labelled recovery internal standards for PCDD/Fs and DL-PCBs.

Category	Congener Name	Concentration (μg/L)
PCDD/Fs	$^{13}C_{12}$-2,3,7,8-TCDD	200 ± 10
	$^{13}C_{12}$-1,2,3,7,8,9-HxCDD	200 ± 10
DL-PCBs	$^{13}C_{12}$-3,2',5,5'-TePCB	5
	$^{13}C_{12}$-2,2,4',5,5'-PePCB	5
	$^{13}C_{12}$-2,2',3',4,4',5'-HxPCB	5
	$^{13}C_{12}$-2,2',3,3',4,4',5,5'-OctaPCB	5

Table S5. The concentrations of PCDD/Fs and DL-PCBs in Chinese mitten crabs and the potential sources (pg/g dw, pg/g ww, pg/L).

Farms	Samples	PCDD/Fs	DL-PCBs	PCDD/Fs and DL-PCB
Shanghai Ruijie Aquaculture Professional Cooperative (RJ)	crab(RJG)	399.93	894.47	1294.39
	sediment(RJCJW)	24032.46	28.65	24061.10

	aquatic plants(RJSC)	202.15	7.62	209.77
	shore plants((RJZW)	73.14	14.55	87.69
	aquaculture water(RJYS)	563.52	11.19	574.71
	feed(RJSL)	2.56	65.81	68.37
Nanxin Village farm(NXC)	crab(NXCG)	340.28	246.51	586.80
	sediment(NXCCJW)	17260.89	11.84	17272.73
	aquatic plants(NXCSC)	146.56	2.12	148.67
	shore plants(NXCZW)	186.02	8.28	194.30
	aquaculture water(NXCYS)	129.43	2.93	132.36
	feed(NXCSL)	1.38	63.38	65.05
Shanghai Mahe Product Professional Cooperative (MH)	crab(MHG)	686.64	502.84	1189.48
	sediment(MHCJW)	35223.59	78.08	35301.67
	aquatic plants(MHSC)	51.88	2.95	54.84
	shore plants(MHZW)	14.19	9.17	23.36
	aquaculture water(MHYS)	341.67	3.57	345.24
	feed(MHSL)	1.94	5.32	7.26
Shanghai Zihao Industrial Development Company Limited(ZH)	crab(ZHG)	20.45	347.31	367.76
	sediment(ZHCJW)	101.98	7.04	109.02
	aquatic plants(ZHSC)	1.19	2.09	3.28
	shore plants(ZHZW)	1.90	1.56	3.46
	aquaculture water(ZHYS)	5.17	11.96	17.14
	feed(ZHSL)	1.16	35.62	36.78
Shanghai Yufeng Aquaculture Professional Cooperative(YF)	crab(ZHG)	63.84	604.32	668.16
	sediment(ZHCJW)	63.84	604.32	668.16
	aquatic plants(ZHSC)	416.68	2.44	419.12
	shore plants(ZHZW)	1.09	1.08	2.17
	aquaculture water(ZHYS)	188.61	1.25	189.86
	feed(ZHSL)	1.32	35.25	35.57
Shanghai Huikang Aquaculture professional cooperative(HK)	crab(HKG)	74.08	442.06	516.14
	sediment(HKCJW)	170.10	23.77	193.86
	aquatic plants(HKSC)	2.31	0.97	3.28

	shore plants(HKZW)	1.44	2.47	3.91
	aquaculture water(HKYS)	5.61	2.69	8.30
	feed(HKSL)	0.74	26.52	27.27

Note: The concentration unit of sediment samples in this table is pg/g dw, the concentration unit of aquaculture water samples in this table is pg/L, and the concentration unit of other samples is pg/g. ww. dw: dry weight; ww: Wet weight.

Table S6. PCDD/F and DL-PCB TEQs in Chinese mitten crabs from fresh markets (pg TEQ/g ww).

Sample Number	PCDD/Fs	DL-PCBs	PCDD/Fs and DL-PCBs
CS1	0.530	0.840	1.37
CS2	0.700	0.130	0.830
CS3	0.280	0.770	1.05
CS4	0.450	1.99	2.44
CS5	1.88	2.34	4.22
CS6	4.08	0.850	4.93
CS7	1.06	1.50	2.56
CS8	0.710	0.920	1.63
CS9	0.940	1.49	2.42
CS10	0.360	1.05	1.42
CS11	0.680	1.22	1.91
DD-1	0.520	0.78	1.31
ZRZ1	0.280	0.380	0.660
ZRZ3	4.36	2.26	6.62
ZRZ4	1.24	1.13	2.36
ZRZ5	0.850	0.930	1.79
ZRZ6	1.18	1.17	2.36
ZRZ7	0.960	0.510	1.47
ZRZ8	0.720	0.280	1.00
ZRZ9	1.14	0.750	1.89
ZRZ10	1.74	0.790	2.53
gZL1	0.290	0.920	1.20
gZL2	0.490	0.600	1.09
gZL3	1.68	1.56	3.24
gZL4	1.90	1.78	3.68
gZL5	3.31	1.49	4.80
gZL6	8.80	2.00	10.8
ZBL1	0.360	1.11	1.47
ZBL2	0.110	0.230	0.330
ZBL3	0.160	0.400	0.560
ZBL4	0.440	0.600	1.05
ZBL5	0.530	1.11	1.64
ZBL6	0.850	0.860	1.70
ZBL7	0.610	0.990	1.60
JD-1	7.11	1.28	8.39
JD-2	4.59	0.720	5.32

Note: ww: wet weight.

Table S7. The total PCDD/F and DL-PCB TEQs in Chinese mitten crabs and the potential sources (pg TEQ/g dw, pg TEQ/g ww, pg TEQ/L).

Farms	Samples	PCDD/Fs	DL-PCBs	PCDD/Fs and DL-PCB
Shanghai Ruijie Aquaculture Professional Cooperative (RJ)	crab(RJG)	11.05	1.00	12.05
	sediment(RJCJW)	18.25	0.04	18.30
	aquatic plants(RJSC)	0.32	0.01	0.33
	shore plants((RJZW)	0.17	0.02	0.19
	aquaculture water(RJYS)	0.91	0.15	1.06
	feed(RJSL)	0.26	0.08	0.34
Nanxin Village farm(NXC)	crab(NXCG)	28.14	1.19	29.34
	sediment(NXCCJW)	17.36	0.07	17.42
	aquatic plants(NXCSC)	0.23	0.01	0.24
	shore plants(NXCZW)	0.27	0.05	0.32
	aquaculture water(NXCYS)	0.27	0.01	0.28
	feed(NXCSL)	0.17	0.05	0.22
Shanghai Mahe Product Professional Cooperative (MH)	crab(MHG)	2.97	1.06	4.03
	sediment(MHCJW)	24.81	0.07	24.89
	aquatic plants(MHSC)	0.10	0.01	0.11
	shore plants(MHZW)	0.08	0.04	0.13
	aquaculture water(MHYS)	0.57	0.01	0.58
	feed(MHSL)	0.22	0.02	0.24
Shanghai Zihao Industrial Development Company Limited(ZH)	crab(ZHG)	0.59	0.64	1.23
	sediment(ZHCJW)	0.47	0.02	0.49
	aquatic plants(ZHSC)	0.07	0.00	0.07
	shore plants(ZHZW)	0.10	0.00	0.11
	aquaculture water(ZHYS)	0.58	0.22	0.80
	feed(ZHSL)	0.09	0.50	0.60
Shanghai Yufeng Aquaculture Professional Cooperative(YF)	crab(ZHG)	7.50	0.60	8.10
	sediment(ZHCJW)	1.23	0.02	1.25
	aquatic plants(ZHSC)	2.77	0.01	2.77
	shore plants(ZHZW)	0.07	0.00	0.07
	aquaculture water(ZHYS)	0.22	0.01	0.23
	feed(ZHSL)	0.08	0.14	0.22
	crab(HKG)	1.70	0.76	2.46
	sediment(HKCJW)	0.43	0.04	0.48

Shanghai Huikang Aquaculture professional cooperative(HK)	aquatic plants(HKSC)	0.04	0.01	0.05
	shore plants(HKZW)	0.16	0.01	0.17
	aquaculture water(HKYS)	0.25	0.01	0.26
	feed(HKSL)	0.05	0.23	0.28

Note: The concentration unit of the sediment samples in this table is pg TEQ/g dw, the concentration unit of the aquaculture water samples in this table is pg TEQ/L, and the concentration unit of other samples is pg TEQ/g ww.

Table S8. The concentrations of PCDD/Fs and DL-PCBs in market crabs (pg/g ww).

Sample Number	PCDD/Fs	DL-PCBs	PCDD/Fs and DL-PCBs
CS1	4.64	398.94	403.58
CS2	6.13	428.82	434.95
CS3	2.86	439.57	442.43
CS4	4.42	745.49	749.93
CS5	10.44	983.44	993.88
CS6	27.03	745.49	749.92
CS7	7.69	563.91	571.60
CS8	5.27	575.59	580.85
CS9	7.76	431.01	438.77
CS10	2.88	410.47	413.35
CS11	10.79	775.72	786.50
DD-1	5.84	315.19	321.03
ZRZ1	2.32	239.75	242.07
ZRZ3	66.71	1204.47	1271.17
ZRZ4	8.59	863.97	872.56
ZRZ5	7.00	677.95	684.95
ZRZ6	9.56	805.63	815.18
ZRZ7	5.97	272.89	278.86
ZRZ8	5.23	223.62	228.85
ZRZ9	9.69	345.16	354.85
ZRZ10	11.62	368.17	379.79
gZL1	2.46	476.11	478.57
gZL2	3.33	719.77	723.10
gZL3	17.76	697.53	715.29
gZL4	27.69	802.91	830.60
gZL5	29.62	422.68	452.30
gZL6	77.67	534.79	612.46
ZBL1	6.36	551.52	557.88
ZBL2	0.81	253.83	254.64
ZBL3	5.60	362.00	367.60
ZBL4	3.36	670.57	673.92
ZBL5	4.83	541.15	545.98
ZBL6	6.61	584.25	590.86
ZBL7	19.00	746.86	765.86
JD-1	237.05	1801.41	2038.46
JD-2	228.44	1934.88	2163.32

Table S9. Ratios of DL-PCBs / PCDD/Fs in farm Chinese mitten crabs and potential sources (pg TEQ/g dw).

District	Samples	PCDD/Fs	DL-PCBs	PCDD/Fs and DL-PCB
Shanghai Qingpu District, China	MHTR	4.86	0.03	4.89
	RJTR	19.01	0.06	19.07
	NXCTR	3.76	0.02	3.78
Shanghai Chongming District, China	YFTR	0.98	0.02	1.00
	HKTR	0.88	0.01	0.89
	ZHTR	0.83	0.04	0.86

Informed Detection Limit (DL) and the percentage of recovery for polychlorido-benzo-p-dioxins/furans (PCDD/Fs) and polychlorobiphenyls (PCBs).

The average detection limits for PCDD/Fs and PCBs in the samples were 3.99 pg and 2.58 pg, respectively. The average recovery rates of $^{13}C_{12}$-labeled PCDD/FS and PCBs toxic counterparts ranged from 25 to 110 percent and 45 percent to 117 percent, respectively, in line with the U.S. Environmental Protection Agency's 1613 and 1668B methods and the National Food Safety Standard - Determination of toxic equivalent of dioxins and their analogs in food (GB 5009.205 - 2013) requirements.

Table S10. Informed detection limit (DL) of the method and the percentage of recovery of each PCDD/F and DL-PCB congener analyzed.

Congener Name	Chinese Mitten crabs		Sediment		Aquaculture Water		Aquatic Plants		Shore Plants		Feed	
	Recovery %	DL (pg)	Recovery %	DL (pg)	Recovery %	DL (pg)	Recovery %	DL (pg)	Recovery %	DL (pg)	Recovery %	DL (pg)
$^{13}C_{12}$-2,3,7,8-TCDF	65.0	2.31	73.8	1.10	57.5	1.88	76.2	2.52	66.7	3.15	65.8	2.73
$^{13}C_{12}$-2,3,7,8-TCDD	64.7	5.28	77.8	2.92	57.3	4.73	90.5	3.95	110	7.77	71.0	6.31
$^{13}C_{12}$-1,2,3,7,8-PeCDF	65.8	3.28	71.4	2.21	41.8	2.63	55.8	2.88	82.2	4.49	65.2	3.79
$^{13}C_{12}$-2,3,4,7,8-PeCDF	58.0	3.27	68.0	2.21	41.8	2.63	69.3	2.88	48.3	4.49	60.0	3.79
$^{13}C_{12}$-1,2,3,7,8-PeCDD	67.5	2.01	74.2	2.14	46.8	1.69	85.0	2.40	87.5	3.52	68.0	2.39
$^{13}C_{12}$-1,2,3,4,7,8-HxCDF	70.2	6.33	84.6	1.00	58.2	5.30	71.8	4.59	54.7	6.78	78.3	7.36
$^{13}C_{12}$-1,2,3,6,7,8-HxCDF	84.3	5.90	84.7	0.933	63.0	4.94	78.2	4.27	62.2	6.32	86.2	6.86
$^{13}C_{12}$-2,3,4,6,8,9-HxCDF	75.8	6.26	80.7	0.992	60.3	5.23	77.2	4.53	57.7	6.70	77.7	7.27
$^{13}C_{12}$-1,2,3,7,8,9-HxCDF	57.0	6.32	60.0	1.00	45.3	5.29	66.5	4.58	91.5	6.77	58.7	7.35
$^{13}C_{12}$-1,2,3,4,7,8-HxCDD	77.5	6.95	75.9	1.21	57.5	6.20	84.0	3.82	64.5	5.11	76.3	7.49
$^{13}C_{12}$-1,2,3,6,7,8-HxCDD	97.8	6.76	89.1	1.18	71.2	6.04	78.3	3.72	65.2	4.97	94.3	7.28
$^{13}C_{12}$-1,2,3,4,6,7,8-HpCDF	55.0	3.78	62.0	0.840	49.0	2.98	47.2	2.22	39.8	3.46	59.5	4.22
$^{13}C_{12}$-1,2,3,4,7,8,9-HpCDF	41.8	4.51	54.6	1.00	35.3	3.54	35.8	2.64	49.8	4.13	47.7	5.03
$^{13}C_{12}$-1,2,3,4,6,7,8-HpCDD	53.7	2.97	63.9	0.772	46.5	3.09	49.3	2.53	60.0	3.45	56.2	3.25
$^{13}C_{12}$-OCDD	30.2	3.67	45.2	1.79	31.7	4.38	34.8	3.82	51.2	6.38	25.3	3.60
$^{13}C_{12}$-3,4,4',5-TePCB	83.0	0.752	63.1	0.694	67.8	0.752	100	1.04	88.5	1.37	78.3	0.913
$^{13}C_{12}$-3,3',4,4'-TePCB	84.8	0.755	62.2	0.702	67.7	0.755	76.0	1.05	78.8	1.37	53.7	0.923
$^{13}C_{12}$-2,3,3',4,4'-PePCB	58.8	2.50	85.5	1.64	62.8	2.15	48.2	3.86	46.8	4.35	90.3	2.19
$^{13}C_{12}$-2,3,4,4',5-PePCB	72.0	2.51	89.9	1.64	64.5	2.15	112	3.87	111	4.36	96.7	2.20
$^{13}C_{12}$-2,3',4,4',5-PePCB	67.8	2.42	78.7	1.59	60.2	2.08	106	3.73	99.2	4.21	86.8	2.12
$^{13}C_{12}$-2',3,4,4',5-PePCB	67.2	2.44	78.0	1.60	59.3	2.10	106	3.77	104	4.25	86.7	2.14
$^{13}C_{12}$-3,3',4,4',5-PePCB	117	1.82	104	1.19	62.8	1.56	107	2.81	103	3.16	51.7	1.60
$^{13}C_{12}$-2,3,3',4,4',5HxPCB	65.5	3.19	87.9	1.94	47.3	2.21	87.5	5.67	64.0	3.96	97.0	2.76
$^{13}C_{12}$-2,3,3',4,4',5'-HxPCB	64.2	3.22	87.6	1.94	60.2	2.23	90.2	5.74	74.3	4.01	97.3	2.79
$^{13}C_{12}$-2,3',4,4',5,5'-HxPCB	65.7	3.04	88.1	1.83	55.5	2.10	98.3	5.42	79.0	3.78	95.3	2.63
$^{13}C_{12}$-3,3',4,4',5,5'-HxPCB	95.0	2.93	87.8	1.76	58.3	2.03	90.0	5.22	78.3	3.65	63.5	2.54
$^{13}C_{12}$-2,3,3',4,4',5,5'-HpPCB	72.7	4.31	67.1	1.81	45.2	2.39	71.2	3.01	75.5	6.60	77.5	1.86

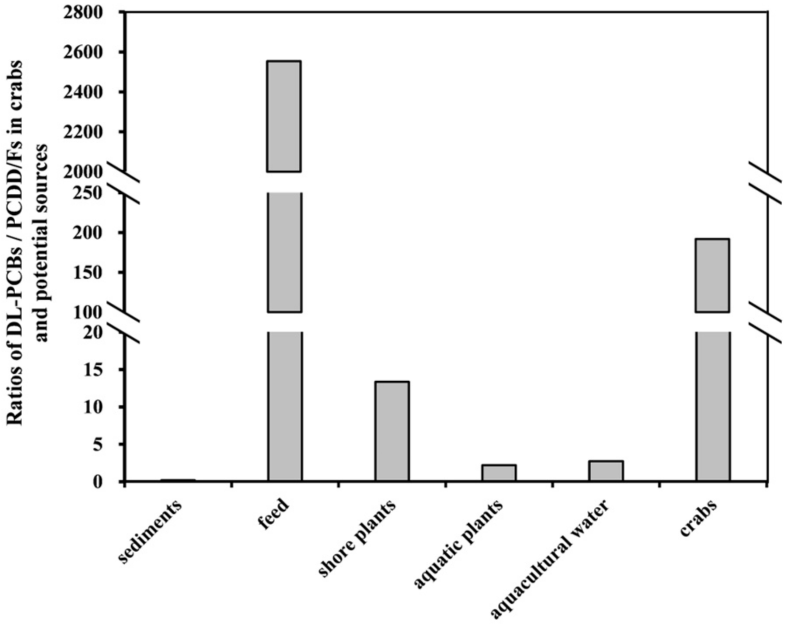

Figure S1. Ratios of DL-PCBs / PCDD/Fs in farm Chinese mitten carbs and potential sources.

foods

MDPI
